# Experiences of General Practitioners and Practice Support Staff Using a Health and Lifestyle Screening App in Primary Health Care: Implementation Case Study

**DOI:** 10.2196/mhealth.8778

**Published:** 2018-04-24

**Authors:** Marianne Julie Webb, Greg Wadley, Lena Amanda Sanci

**Affiliations:** ^1^ Department of General Practice Melbourne Medical School University of Melbourne Parkville Australia; ^2^ School of Computing and Information Systems University of Melbourne Parkville Australia

**Keywords:** adolescent, primary health care, primary prevention, health behavior, quality improvement, telemedicine

## Abstract

**Background:**

Technology-based screening of young people for mental health disorders and health compromising behaviors in general practice increases the disclosure of sensitive health issues and improves patient-centered care. However, few studies investigate how general practitioners (GPs) and practice support staff (receptionists and practice managers) integrate screening technology into their routine work, including the problems that arise and how the staff surmount them.

**Objective:**

The aim of this study was to investigate the implementation of a health and lifestyle screening app, Check Up GP, for young people aged 14 to 25 years attending an Australian general practice.

**Methods:**

We conducted an in-depth implementation case study of Check Up GP in one general practice clinic, with methodology informed by action research. Semistructured interviews and focus groups were conducted with GPs and support staff at the end of the implementation period. Data were thematically analyzed and mapped to normalization process theory constructs. We also analyzed the number of times we supported staff, the location where young people completed Check Up GP, and whether they felt they had sufficient privacy and received a text messaging (short message service, SMS) link at the time of taking their appointment.

**Results:**

A total of 4 GPs and 10 support staff at the clinic participated in the study, with all except 3 receptionists participating in the final interviews and focus groups. During the 2-month implementation period, the technology and administration of Check Up GP was iterated through 4 major quality improvement cycles in response to the needs of the staff. This resulted in a reduction in the average time taken to complete Check Up GP from 14 min to 10 min, improved SMS text messaging for young people, and a more consistent description of the app by receptionists to young people. In the first weeks of implementation, researchers needed to regularly support staff with the app’s administration; however, this support decreased over time, even as usage rose slightly. The majority of young people (73/87, 84%) completed Check Up GP in the waiting room, with less than half (35/80, 44%) having received an SMS from the clinic with a link to the tool. Participating staff valued Check Up GP, particularly its facilitation of youth-friendly practice. However, there was at first a lack of organizational systems and capacity to implement the app and also initially a reliance on researchers to facilitate the process.

**Conclusions:**

The implementation of a screening app in the dynamic and time-restricted general practice setting presents a range of technical and administrative challenges. Successful implementation of a screening app is possible but requires adequate time and intensive facilitation. More resources, external to staff, are needed to drive and support sustainable technology innovation and implementation in general practice settings.

## Introduction

### Screening Young People for Mental Health Disorders and Health-Compromising Behaviors

A range of mental health disorders and health-compromising behaviors emerge during adolescence and young adulthood, compromising current and future health and well-being [1]. Best practice guidelines recommend regular screening of young people for health and lifestyle issues in primary care to detect and intervene with problems early [2,3]. Screening increases appropriate referrals to specialty care [4] and the provision of health education materials [5] and may improve health outcomes [6]. However, many young people face barriers to disclosing health risks, and many clinicians run out of time or are concerned to ask about sensitive issues, especially if not directly related to the presenting issue [7,8]. Technology-based screening has the additional advantages of being engaging and efficient [9].

Our own prior research with 85 young people found that using a health and lifestyle screening app increased disclosure, patient-centered care, and preparedness of young people attending general practice. It created scope to address unmet health needs, without negatively affecting indicators of youth-friendly quality care, such as privacy and trust in their general practitioner (GP) [10]. In our study, we found that only 14.4% (30/209) of the eligible patients did not use the app, either because they did not want to use the app (n=24) or for no provided reason (n=6) [10]. We also found that young people rated this screening technology as highly acceptable and very easy to use, and most of them wanted to use the screening app regularly as part of their routine care with their GP [10]. However, despite the demonstrated benefits and need, the rate of using digital technology beyond a basic electronic health record in primary care settings is low [11,12].

### Implementation of Interventions to Improve Health in Primary Care

Translating evidence and clinical guidelines into routine practice is typically slow and erratic and can result in suboptimal care and patient exposure to unnecessary risks [13]. A challenge for knowledge translation into primary care is the complexity of the setting, where multiple agents, such as patients, GPs, and practice support staff (receptionists and practice managers), are interacting at and across multiple levels, including organizationally and individually, and not always in a predictable manner [14]. The fit between an intervention and these dynamic contextual factors has been found to be critical in determining the success of an implementation in shortening the evidence-to-practice gap [15].

The Medical Research Council, which coordinates and funds medical research in the United Kingdom, recommends a staged and iterative process for the development, evaluation, and implementation of complex interventions to improve health [16,17]. Complex interventions comprise several components which interact and do not necessarily relate linearly or predictably [16]. An iterative approach to tailoring interventions, repeatedly reflecting on results and refining, is particularly appropriate for the implementation of health technology, a complex intervention which is influenced by underlying and multifaceted interrelated technical, social, and organizational factors [18].

Reflecting the growth of implementation research, multiple theories exist to explain how and why implementation succeeds or fails [19]. Normalization process theory (NPT) provides a particularly appropriate theoretical lens to investigate the implementation of a screening technology in the primary care setting and has been widely used [20-22]. Unlike most other theories, NPT accounts for the important social aspects of the implementation of health technology, including the work required to make sense of the technology and its effects on roles and responsibilities [20]. NPT describes how complex practices and technology innovations become embedded and integrated into health care settings [23,24] and posits that implementation is operationalized in social contexts, including work settings, through 4 key mechanisms: coherence, how participants make sense of an intervention; cognitive participation, commitment and engagement by participants; collective action, the work participants do to make the intervention function; and reflexive action or how participants reflect in appraisal of the intervention after it has been in use.

### Experience of Clinicians and Support Staff in Implementing Technology-Based Screening for Young People in Primary Care

Health care providers have reported that using a technology-based screening tool facilitates identification of and communication about risky behaviors [9], while pediatric primary care providers report that these tools improve visit organization and efficiency [25]. Little is known about how GPs integrate screening technology into the context of routine clinical care. One study found that using a quality improvement framework resulted in increased rates of comprehensive screening and counseling for adolescents [26], but this study was conducted in predominantly pediatric settings and did not use technology. In the United States, where young people may attend planned well-child consultations, which devote time to screening and intervention, less than 50% do so [27,28], which is why it is important to investigate whether primary care providers are able to use a technology-based screening tool during consultations in which young people present acutely with any health issue in a primary care setting. This investigation is even more pertinent for generalist primary care services which are a gateway to specialist services, such as general practice in the United Kingdom, Australia, New Zealand, and Canada, which treat patients of all age groups and thus have to balance patient care processes for each group.

In addition, as the interface between the health care system and the community [29], support staff are typically the staff responsible for administering screening tools required to be completed by patients before the consultation. Support staff have highly demanding and complex roles [30], yet they also report having limited agency [31], which is known to impede implementation in primary care [15]. Despite their central role, there is a paucity of studies that explore the needs and experiences of support staff in implementing technology-based screening tools.

### Aim of This Study

The aim of this study was to investigate the implementation of a codesigned health and lifestyle screening tool (“app”), Check Up GP, for young people in an Australian general practice. We used NPT to explore both the experiences of support staff in administrating an app to young patients and how GPs integrated the screening tool within their routine care of young people attending a general, rather than youth-specific primary health care setting. The codesign process for Check Up GP has been described in a previous conference publication and included input from young people, parents, support staff, and GPs [32].

## Methods

### Study Design

We conducted a 2-month in-depth implementation case study of Check Up GP in one general practice clinic, with methodology informed by the action research approach. Case study research is a particularly useful method for investigating complex social interventions and settings in health care [33], including technology-related innovations [34]. Action research aims to involve stakeholders in the implementation of change and generation of knowledge in a real-world setting through a cyclical process of collecting, feeding back, and reflecting on data [35,36]. A Plan-Do-Study-Act (PDSA) framework was used to help the clinic structure the iterative improvement of Check Up GP and its administration in rapid quality improvement cycles [37]. The PDSA framework has the advantage of being recommended to general practices to use in quality improvement activities by the Royal Australian College of General Practitioners, the peak accreditation body for GPs in Australia [3]. The 2-month length of the study was reached by agreement with the clinic before the commencement of the study. The study received ethics approval from the University of Melbourne (Ethics ID #1544281).

### Recruitment

The clinic was recruited through the Victorian Primary Care Practice-Based Research Network, managed by the Department of General Practice at the University of Melbourne. The 4 GP principal owners and all support staff participated in the study. The support staff consisted of a practice manager, a reception coordinator, and 8 receptionists, with all except 3 receptionists participating in the final interview.

The site chosen met 4 key criteria for selection in this case study. We wanted a traditional group practice that is run by GPs rather than a corporate chain practice, a community health center, or a bulk billing clinic (services covered by Australia’s Medicare system of free universal health insurance), as traditional group practices form the majority of primary care practices within Australia. We wanted a practice that was interested in young people and saw enough young people in the age group of 14 to 25 years but was also a generalist practice caring for patients from cradle to grave. We wanted a practice that was comfortable with using technology and willing to work on integrating it but was not highly tech savvy as most of the practices in Australia have only a basic level of tech use (most only use technology for appointments, pathology, prescriptions, and billing [11,38]). We needed a site where most patients would be able to read and understand English, the language used in this prototype of the tool.

### Case Study Context

Using the macro, meso, and micro typology developed by Bronfenbrenner [39], the context of the case study is described below.

#### Macro Level Contextual Factors

The clinic is located in an inner urban suburb of Melbourne, Australia, just over 10 km east of Melbourne’s central business district. It is in a Local Government Area (LGA) ranked in the 98th percentile in Australia’s Index of Relative Socioeconomic Disadvantage [40], indicating a relative lack of economic and social disadvantage. Compared with the whole of Australia, the LGA has a similar mean age (LGA=37.7 years, Australia=37.2 years) [41] and proportion of young people aged 15 to 24 years (LGA=27,243 / 177,361; 15.36% [41], Australia=3,172,058 / 24,206,201; 13.01%) [42]). However, the LGA has a higher median income (LGA=Aus $56,451, Australia=Aus $46,854) [41]) and proportion of completion of secondary education (LGA=74.8%, Australia=51.8%) [41]. The unemployment rate of the LGA is similar to the national average (LGA=4.5%, Australia=5.6%) [41], though the proportion of people born overseas is lower (LGA=30.9%, Australia=35%).

#### Meso Level Contextual Factors

Established in 1902, the clinic is in a freestanding converted house located in a residential area at the edge of a local shopping village. Open 365 days per year, it is a large clinic with 12 GPs (4 principal owners and 8 GP employees or associates, totaling a full time equivalent (FTE) of 7.2), 5 registered nurses (FTE 2.15), a practice manager (PM; FTE 1.0), a reception coordinator (RC; FTE 1.0) and 8 receptionists (FTE 4.8). Colocated at the clinic are a podiatrist, dietitian, and a pathology service. It is a mixed private and bulk-billing practice with approximately 30% of patients being Health Care Card holders (the Health Care Card is provided by the Australian Government to those on certain government benefits, entitling the holder to concessions on the cost of health services and medicines). Those younger than 16 years, and pensioner and concession card holders (provided by the Australian Government to those receiving old age, carer, or disability pensions) are only bulk billed between 10:00 AM and 4:00 PM from Monday to Friday, though the GPs have the discretion to bulk bill during other times. As a general practice, every GP sees patients across the life span, including young people, and between 46 and 100 young people aged 14 to 25 years attend the clinic each week.

#### Micro Level Contextual Factors

The clinic uses a commercial clinical electronic health record software package for appointments and patient files. They have recently started using the software’s automated SMS text messaging (short message service, SMS) function to send bulk appointment reminders and health care recall messages to patients. Organizing these SMSs is the responsibility of one support staff member (the reception coordinator); so other receptionists neither use nor are familiar with using this SMS functionality. Before this study, neither the GPs nor support staff had prior experience with introducing new technology or apps into their work, though they were interested in the potential of technology to improve their work. Apart from new patient forms, receptionists did not have experience with asking patients to complete any offline or Web-based screening or other preappointment forms. The clinic does not have wireless Internet in the clinic, and new patient information is collected via paper forms and then transcribed by receptionists into their software. The GPs are used to checking clinical information, such as pathology results, before seeing their patients, though they do not typically navigate away from their clinical software to other websites during consultations.

The 4 GP principal owners are usually the instigators of change, and it is the PM’s responsibility to show support and sustain momentum. The principal owners oversee the clinical function and are responsible for the GPs and nurses. The PM has complete autonomy over administration: receptionists report to the RC, and the RC reports to the PM. The PM and principal GP owners together form the senior management team. Clinical meetings involving all doctors are scheduled once in every 6 weeks, administration meetings (including one principal GP) are scheduled once in every 3 months.

As reported previously [10], the GPs rated highly their enthusiasm for seeing young people and their knowledge and confidence in consulting and communicating with young people and their confidence in exploring issues beyond the presenting problem.

### Measures

At the beginning of the study, GPs and support staff participants were asked to rate their technology adoption at home and at work on a scale (from 1=slow adopter to 5=innovator). At the end of the implementation, semistructured interviews and focus groups were conducted in-person with GPs and support staff by MW. The interview guide (Multimedia Appendix 1) was informed by the 4 core constructs of NPT to explore participants’ experience of implementing Check Up GP. During the implementation period, researchers recorded the number of times that we needed to provide support to staff on using the app. Young people completed an exit survey immediately after their consultation. Results on what young people thought about using the app as part of their routine health care and the impact on their experience of care are reported in a previously published paper [10]. In this paper, we report on new data from young people concerning the implementation of the app: where they completed Check Up GP, if they received an SMS with a link to Check Up GP before attending the practice, and if they felt they had sufficient privacy.

### Intervention

The content and design features of Check Up GP are described in detail elsewhere [10]. In brief, the app consisted of 2 parts: the questionnaire answered by patients and the GP summary report. The questionnaire was adapted from the HEEADSSS (Home environment, Education and employment, Eating, peer-related Activities, Drugs, Sexuality, Suicide and depression, Safety from injury and violence) preventive health framework for interviewing adolescents [43,44], covering the range of mental, physical, and social issues and behaviors commonly experienced by young people. Questions from validated screening instruments were included to capture depression (Patient Health Questionnaire-2) [45], generalized anxiety disorder (Generalized Anxiety Disorder-2 scale) [46], eating disorders (Sick Control One stone Fat Food questionnaire) [47], and substance-related risks and problems (Car Relax Alone Forget Friends Trouble test) [48]. Youth responses to the questions in the app were immediately conveyed to their GP via a secure website. This summary highlighted areas of concern and strengths as well as tips on youth-friendly consultations and suggested actions to take on areas of concern, including referral options, information, and resources.

Receptionists were trained to inform young patients aged 14 to 25 years of participating GPs that Check Up GP was available when they phoned the clinic to take an appointment. The receptionist then created a flag in the clinical software signaling the reception coordinator to send the patient a link to Check Up GP via an SMS message (sent from the clinical software). When a young patient arrived at the clinic for their appointment, receptionists asked the young person if they had received the SMS; if not, the young person was given an Apple iPad and asked to complete the tool in the waiting room before their consultation. Once the tool was completed, the receptionist notified the GP via the clinical software that the GP summary report was available for checking.

### Procedure

At the start of the study period, all participating GPs and support staff met with the researchers (MW, GW, LS) and were given an overview of the study. In accordance with the iterative and participatory nature of the action research approach, we worked closely with staff during the intervention period to facilitate improvements to the process in rapid quality improvement cycles. A researcher was always present in the practice’s waiting room during the intervention to collect exit surveys from young people immediately after the consultation and to provide implementation support to staff if requested. Also, at the beginning of the study period, because the clinic did not have wireless Internet, we set up a wireless dongle in the waiting room to enable patients to complete Check Up GP in the clinic and for the results to be immediately available to the GPs.

Young patients were recruited in the waiting room upon arrival to the clinic. If they were seeing a participating GP and aged 14 to 25 years, the researcher provided them with information about the study. If they agreed to participate, the young patient was asked to take a form into their consultation for their GP to assess their eligibility to participate in the study. Patients were excluded from the study if their GP assessed that their patient was very unwell (eg, vomiting, weak, psychotic), unable to read or speak English, or if they were younger than 18 years and not a mature minor [49]. The patient handed the form back to the researcher on returning to the waiting room. Immediately after their consultation, each youth participant completed the short exit survey on a tablet in the waiting room with consent provided at the start of the survey.

During the 2-month implementation phase, the researchers communicated regularly with the PM and met twice with the GPs during a 30-min lunch break, using the PDSA framework as a structure for quality improvement discussion. At these meetings, we presented a summary of results from youth exit surveys to that date, including rates of youth-rated patient-centered care and disclosure. We then asked staff to reflect, identify, and resolve challenges or problems with either the implementation or the tool itself. These meetings also provided an opportunity to share feedback on initial findings, ensure the practice remained engaged, and celebrate successes. On the basis of staff needs, changes were made to the tool and how the tool was implemented by staff. We were unable to meet the receptionists as a group due to lack of availability, so ad hoc sessions were run with individual receptionists during quiet times in the waiting room. We also distributed a number of supporting documents to receptionists: a Frequently Asked Questions document to clarify and reiterate procedures, such as recommended phrases to use when informing young people about the tool and a summary of anonymized results from young people’s exit surveys, including ratings of disclosure and patient-centered care.

The clinic received Aus $2000 for their involvement (Aus $1000 at the beginning and another Aus $1000 at the end of the study), and each support staff received an Aus $100 gift voucher at the end of the study in recognition of the additional work required of them to participate in the study. Involvement in the study was also an approved activity of the Quality Improvement and Continuing Professional Development program for GPs through the Royal Australian College of General Practitioners.

### Analysis

Interviews with GPs and support staff were audio-recorded and then transcribed verbatim by a professional transcription service. The first author listened to all interviews to ensure the transcripts accurately reflected the audio recordings. Transcripts were coded by the first author using NVivo 11 (QSR International, Melbourne) software. The second and third authors used this coding framework to independently code 2 transcripts: an interview with a GP and an interview with a receptionist. All authors then met to compare codes, revising the coding structure as required. The first author subsequently recoded all transcripts using the updated coding framework, before conducting a thematic analysis [50], with themes mapped to the corresponding core NPT constructs. The authors, of whom one is also a practicing GP (LS), met regularly to discuss emerging themes and resolve any differences until a final set of themes was agreed upon. Descriptive statistics were used to analyze results from the youth exit survey (where young people used Check Up GP, whether they received an SMS with a link to it before attending the practice, and whether they felt they had sufficient privacy). Ratios were calculated of both usage and support provided to total eligible patients.

## Results

### Participant Characteristics

All 4 participating GPs were male, with three aged between 45 and 54 years and one aged between 55 and 64 years. All the 10 support staff who consented and participated were female. Of the 7 support staff who completed the demographic survey and participated in the final interviews, 2 were aged between 25 and 34 years, 1 was aged between 35 and 44 years, 2 were aged between 45 and 54 years, and 2 aged between 55 and 64 years. Compared with support staff (n=10), GPs (n=4) rated their technology adoption lower (between 1=slow adopter and 5=innovator) in both work and home settings (GP work mean=2.75, SD=1.26, GP home mean=3, SD=1.41, support staff work and home mean=3.9).

The characteristics of the young people who used Check Up GP are reported in detail in a previously published paper [10]. Briefly, of the 85 young participants who used the app, 54% (46/85) were female and the remaining 46% (39/85) were male. The mean age of youth participants was 19.9 years (SD 3.32). Just over half of young people (51/85, 59%) did not have a parent or guardian with them at the clinic.

### Implementation Activities

**Figure 1:**
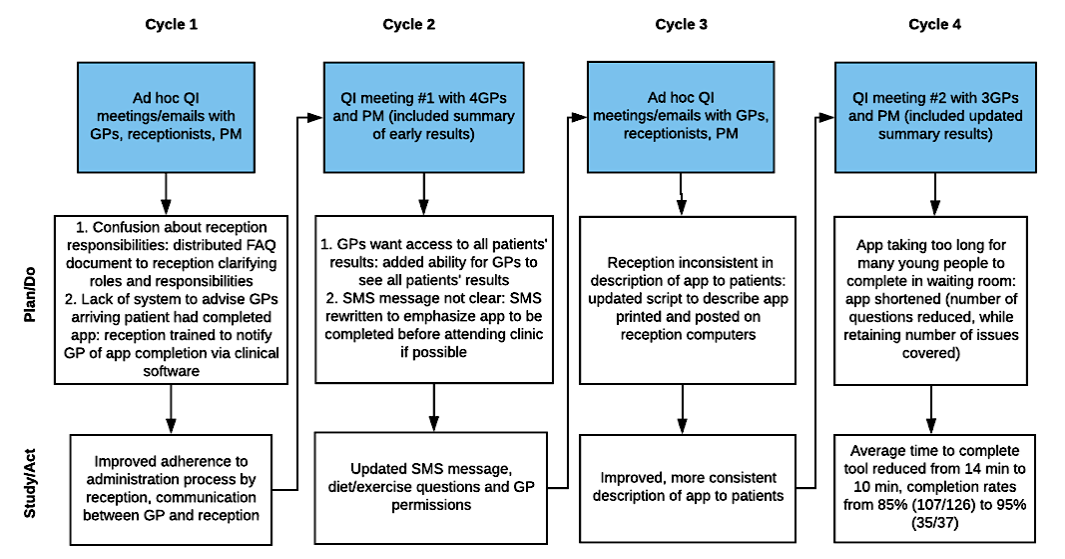
Iterative cycles of quality improvement (QI) activities and results using the Plan-Do-Study-Act framework by general practitioners (GPs), practice manager (PM) and receptionists during the implementation of Check Up GP. FAQ: frequently asked questions; SMS: short message service.

As shown in Figure 1, during the implementation period, the design of Check Up GP was improved iteratively after 4 major PDSA quality improvement cycles in response to the needs of GPs and support staff. Changes that were made to the tool and its administration resulted in the following: a reduction in the average time taken to complete Check Up GP from 14 min to 10 min, an updated SMS message for young people, and a more consistent and improved description of the app for receptionists to use when speaking with young people.

### Rates of Youth Usage and Support Provided to Staff

Table 1 shows, in each week of the 2-month implementation period, the usage of Check Up GP by young patients, the number of times research assistants helped GPs and support staff with administering Check Up GP or technology problems, and the ratios of both usage and support to total eligible patients. The ratios show that, although the proportion of usage of Check Up GP by eligible patients increased slightly throughout the study period, the proportion of support required by staff to eligible patients declined.

### Postimplementation Staff Interviews and Focus Groups

Our findings from the staff interviews and focus groups conducted at the end of the intervention are structured here within the 4 core constructs of NPT.

**Table 1:** Check Up GP rates of patient usage and support provided to staff and ratios of usage and support provided to total number of eligible patients.

Patient usage and support provided to staff		Week 1^a^	Week 2	Week 3	Week 4	Week 5	Week 6	Week 7	Week 8^a^
Patient usage		7	18	10	9	13	6	12	9
Number of times support provided to staff		19	19	14	11	6	3	5	5
**Total number of eligible patients**		22	31	30	21	25	12	25	17
	Usage: total eligible patients	0.3	0.6	0.3	0.4	0.5	0.5	0.5	0.5
	Support: total eligible patients	0.7	0.6	0.5	0.5	0.2	0.2	0.2	0.3

^a^Week included 1 day of public holiday when only one GP worked and clinic opened for 4 hours instead of normal 12 hours.

#### Coherence

Both support staff and GPs could identify the purpose of Check Up GP and the potential benefits for young people using the tool. They felt that a key benefit of the tool was that young people’s comfort with technology would facilitate disclosure of health and lifestyle issues:

I think it’s a user-friendly way for the young generation, who are so comfortable with electronics, to bring up topics that they don’t particularly feel comfortable doing face-to-face…it means the doctors find out more than they probably would have.

PM

This is an amazing opportunity to speak to young people about all sorts of stuff that they would never ever have spoken about before.

GP 4

Although Check Up GP was viewed as being beneficial, GPs also speculated about 2 potential problems with using it. One GP felt that young people’s responses may be impetuous or fleeting and not necessarily provide an accurate reflection of their health and lifestyle:

You’ve got the impulsivity of the kids as well coming in [saying] “Oh, this is how I feel this morning. I don’t actually feel that bad, it’s just what I wrote because I got out of the wrong side of bed this morning.”

GP 4

The experience of another GP was that not all his patients were receptive or had a positive experience of using Check Up GP:

There was a small number who didn’t really think it was very helpful or there were some who actually openly thought it may not be good but most were very open to it.

GP 1

Staff could make sense of the tool by comparing it with similar existing or previous interventions:

We do the same thing with 45 to 49 year old health check...and it’s a little bit similar isn’t it.

GP 1

We do surveys and things all the time.

Receptionist 1

#### Cognitive Participation

All staff interviewed felt that implementing Check Up GP was part of their existing job description and responsibilities. Support staff reflected that they were regularly required to integrate new systems or processes, which could originate from within the clinic, such as a new telephone system, or externally, such as new billing requirements from Medicare (the Australia Government’s public health insurance scheme). The staff’s understanding of the purpose of Check Up GP also seemed to facilitate their acceptance and buy-in of using the tool. One of receptionists stated:

It’s just part and parcel of the job. I think we should be expected to do it. It’s for patients and we’re here to provide a service, so yeah.

Receptionist 5

Cognitive participation was evident in the way in which staff took initiative and invested time to improve the administration of Check Up GP throughout the implementation period. For example, it took some time for receptionists to normalize the correct script for describing Check Up GP to young people. One receptionist said:

You [researcher] spoke to [the practice manager] at one point; you said let’s get the receptionist to say this [how the app was described to the patient], I cut that out and I put it on my keyboard, and I stuck it down with sticky tape.

Receptionist 2

#### Collective Action

We found that collective action was the biggest challenge for the implementation of Check Up GP. Administering the app presented significant additional work for support staff, who were already busy and had limited time to learn and integrate the new process into their work. For instance, the PM said:

There was lot of stress from them about an additional task. They’re constantly multi-tasking, there are a number of lines on hold, doctors who want attention right now. So it is a busy environment.

PM

The task of sending the link to Check Up GP by SMS, one of the core requirements of using the tool, was particularly time-consuming, creating a substantial amount of additional work for the RC. Unlike the automatic patient appointment reminders that are sent by the clinical software, the Check Up GP SMSs were not integrated with clinical software and had to be manually sent for each patient. In the words of an RC:

You had to look at the upcoming appointments, check who fit into the category. Then you go into another screen on the computer and write up the message, and make sure that you send them to all. And then you go back into the name and you put under the alert that you note that you have send them the SMS…[The time it takes] depends how many patients you had on the day…at one stage we had about 12 that required a bit, that took at least almost 40 minutes to do.

RC

Lack of time was also a factor that influenced GPs use of Check Up GP. Using the app inherently added additional time to a consultation and GPs felt they had to rush to address all issues raised. For one GP who always ran to schedule, there was often not enough time for patients who did not arrive early to complete Check Up GP in the waiting room as this GP was not prepared to run even a little over time or to wait for young people to complete Check Up GP. Another GP felt that it was feasible to continue to use Check Up GP as part of young people’s routine care, though not at certain times, such as on weekends when only one GP works or during very busy periods.

Perhaps reflecting the time pressures and additional work, staff compliance with using Check Up GP was not always consistent, especially at the start of the implementation period. It took time for receptionists, particularly those working part-time, to understand what was required and to normalize the procedure. One of the receptionists stated:

I found in the beginning it was quite overwhelming because I was here part-time...when you first started it, I’m thinking, “What is going on here?”

Receptionist 1

Inconsistent compliance by receptionists in administering Check Up GP required intervention by a GP at one point in the intervention:

A couple of times we had to say [to receptionists]: “look, do you know what, this is not an optional thing, this is actually what we’ve chosen to do and it’s important and this is part of the job.”

GP 1

As well as time pressures, a lack of feedback was a factor that influenced the receptionists’ collective action. One receptionist stated:

I would have liked a bit of feedback [from the GPs] with whether they felt it was successful or not…because if they don’t think it’s good then why would we really do it...even if it was an email to say that this is the feedback that we found...or “Thank you, receptionist, for doing a great job.”

Receptionist 1

One of the biggest facilitators of collective action was the support provided by researchers throughout the implementation period. This support was often practical, such as answering questions or reminding reception on the correct process of administrating Check Up GP. As one receptionist said:

Your staff were there, so if we did have issues and it got too busy we just go “hey, come out here” and “can you help.”

Receptionist 2

As well as practical support, staff appreciated the feedback provided by researchers about the impact that using the tool was having on young people, as evidenced in improved ratings of disclosure and patient-centered care for those using Check Up GP compared with a treatment-as-usual group:

It was good to have feedback from what we’re participating in...it’s good to know that that was helpful.

Receptionist 3

#### Reflexive Monitoring

GPs felt Check Up GP had the potential to transform the experience of care, by expanding young people’s understanding of the scope of what their GP can help them with. One of the GPs said:

This very tool itself might give them the confidence to appreciate what is possible in the consultation...this is one of the truly significant advances, I think, in adolescent health.

GP 4

While the GPs were mostly positive about the impact of using Check Up GP on their care of young people, one GP reported that there were times when he felt ill-prepared to deal with an issue raised. There was an acknowledgment from GPs that further training was needed to equip them with the skills to help patients with raised issues:

There were a few times I didn’t know what to do with information...the kid who ticked “I often feel alone,” okay, that’s sad…it made me feel uncomfortable, that’s not my kind of strength.

GP 3

Upskilling might be useful for us to deal with specific problems...that would be a way of making it work because without the skillset in the GPs, it’s all very nice but it isn’t going to go anywhere.

GP 4

GPs were able to reflect on the impact of using Check Up GP on their care of young people. One key advantage was Check Up GP provided a reason to ask for time alone when young people were attending with a parent. One of the GPs stated:

It made it easier to deal with the parents, it made it easier to throw them out of the room...It’s not like, “oh, we’ve been talking about your presenting problem, and now I’m going to ask the parent to leave for some vague, nebulous kind of opportunistic waste of time that you don’t have.” It was like it forces you to do it.

GP 3

Both receptionists and GPs observed that there was often a lack of privacy using Check Up GP for young people attending the clinic with a parent. This lack of privacy had the potential to undermine the purpose of the tool, as is evident from the following statement:

I found some of the mothers were quite intrusive. The kids were sitting there trying to do it...Like they’re not going to answer something candidly, tick “Yes, I take party drugs” or “Have unprotected sex” with mum sitting on top of them, are they? So I felt that that might have influenced some of the answers to be done not honestly for the sake of offending their parents.

Receptionist 5

Both support staff and GPs felt that the implementation improved with time, as the process became embedded:

We probably had a little bit more understanding as it went on what was happening. But I mean not that we weren’t explained well enough, I think it’s just getting used to doing that role.

Receptionist 3

I think it’s great. I think it does take a bit of time to get up and running with it, some months. So if you want to introduce it, you’ve got to be prepared to put that in.

GP 4

For sustained use both support staff and GPs felt the tool would need to be fully integrated and automated into their clinical software. One of the GPs said:

I think because it was a trial that it didn’t really worry us. I think, moving forward, I would like to see the information in the patient’s file, because otherwise to then go looking, hunting for it amongst a scroll of names is going to be really difficult...it’s not going to be functional.

GP 4

Finally, despite the implementation challenges, GPs expressed a desire to continue using Check Up GP regularly with their young patients. Indeed, when the app was removed at the end of the study one GP reflected on how it would have saved time to assess a recent patient, who presented with a psychological issue:

Certainly, the assumption is it worthwhile putting to routine [use]...it’s hard to know how often you should be doing it but probably...at least [young people] being offered it every couple of years would be fabulous.

GP 1

Another GP said:

Yes [it would have saved me time] because they’re the questions I want to ask now. It would have been good if they [responses] were there and selected and she would have told me [about her] sexual health and all that.

GP 3

### Implementation Results From Young People

When asked where they completed Check Up GP, almost all (83/85, 98%) youth participants reported completing it in just one location. The majority of young people (73/87, 84%) completed Check Up GP in the waiting room, while 13% (11/87) completed it at home. Only a few completed it at work (2/87, 2%) or school/university (1/87, 1%). Of those who answered the question, 44% (35/80) of young people reported receiving an SMS from the clinic, 41% (33/80) did not receive an SMS, while 15% (15/80) could not remember. A large majority (79/85, 93%) of young people felt they had sufficient privacy completing Check Up GP, although the remaining 7% (6/85) felt they did not have enough privacy.

## Discussion

### Principal Findings

The aim of this study was to investigate the implementation of a codesigned health and lifestyle screening app for young people attending general practice for routine care. We conducted an in-depth implementation case study of Check Up GP, using a methodology informed by action research and NPT as a framework to guide our analysis. Overall, we found that, with appropriate time and intensive support from researchers, it is possible to implement a health and lifestyle screening app into the routine care of young people attending general practice.

One of the key challenges for GPs was the collective action, or operational work, required to implement Check Up GP. GPs expressed concern that they did not have time in routine consultations to sufficiently address all issues identified through Check Up GP, in addition to the presenting acute health issue. Given that the duration of a standard primary care consultation is approximately 10 min [51,52], this concern is understandable. There was also a concern from one GP that he did not always feel equipped to address some issues raised. Interestingly, these concerns were not reflected in young people’s experience of using Check Up GP. As reported previously, the majority of young people felt that their GP addressed the issues raised in Check Up GP either “quite a bit” or “very much [10].” It may be that young people were satisfied that any issues identified by the app were acknowledged and could be followed up in future consultations and did not expect an immediate lengthy discussion. This finding suggests that the perceptions of GPs are not necessarily reflected in the experience of patients and that patients offer important and unique insights into implementation.

Similar to GPs, support staff had high coherence and cognitive participation with the intervention, recognizing Check Up GP’s potential to improve the care of young people. However, it took time for them to normalize the administration of the app into their hectic day-to-day work routine, and improvements did not happen in a linear way, in keeping with the nature of complex interventions [16,53]. This normalization was impeded by the substantial additional time required by support staff to flag eligible patients in the clinical software, manually send individual SMSs, and then notify GPs when the tool was complete and the summary report available for viewing. Given this added work, it is not surprising that only 44% (35/80) of young people reported receiving an SMS with a link to the screening tool before arriving at the clinic. Although it was outside the scope of our study, automating this process and integrating the app within existing clinical software would facilitate implementation.

Not every issue was resolved by the end of the study. This suggests that full implementation, where the intervention is self-sustaining, was not achieved. Despite this, the implementation did improve over the 2-month study period, with youth relative usage remaining stable even as support required decreased. That full implementation was not achieved within the 2-month study period is not surprising given that successful implementation of new technology in primary care may take years [54,55]. It is also important to note that success in implementation is a dynamic and multidimensional concept, evolving over time [56,57]; so though using the app was not yet fully integrated into participants’ workflow, it was successful in terms of its acceptance, feasibility, and effectiveness.

An important facilitator for the implementation of Check Up GP, enabling collective action, was the intensive support provided by researchers onsite throughout the implementation period. Both support staff and GPs appreciated having a researcher at hand to troubleshoot issues that emerged after the clinic started using Check Up GP and to improve the implementation through rapid quality improvement cycles. This process enabled the intervention to better fit the context of a very busy primary care service where additional time to manage adoption of a new process that is a departure from usual process is slim. NPT does hold that implementation is easier when the new process blends easily with routine [24]. Dealing with these sorts of contextual issues is an important requirement in facilitating implementation in primary care [15]. Having the support of the researchers onsite meant that instead of having to pause the implementation for days or even weeks, most issues were able to be promptly investigated and resolved. Our role in facilitating the implementation of Check Up GP was, in essence, that of a practice facilitator. Practice facilitators assist primary care practices with coordinating quality improvement activities and building capacity for those activities [58]. Practice facilitation has been shown to improve the adoption of evidence-based practices in primary care [59]. More research is needed to investigate the role of practice facilitators in helping primary care practices adopt new technology, but it does appear from our study that in very busy practices juggling competing demands, implementation is more effective if a facilitator is dedicated to the task.

Another factor that facilitated the extent of Check Up GP’s implementation was the context of our case study. The clinic did not have prior experience or systems to support the introduction of Check Up GP. However, it is likely that being located in an area of socioeconomic advantage provided greater scope for innovation compared with practices located in less-advantaged areas. As described by Hart’s Inverse Care Law [60] and supported in an analysis of Australian general practice data [61], people located in more-advantaged areas tend to receive longer consultations than those in more-disadvantaged areas, even though the frequency of care is similar. In addition, the business model of our practice may have facilitated innovation. Being a mixture of bulk billing and copayment may provide the practice with more money and time to see patients and scope to innovate compared with bulk billing only practices, which typically have very limited time and resources. Also, unlike corporate-owned practices, our clinic was privately owned by four of the practicing GPs, meaning implementation was not held up or dependent on approval of offsite management.

Strong organizational leadership and management support are important factors in the effective implementation of health technology [18,62]. Apart from the initial project orientation session run by the researchers, support staff and GPs did not manage to meet as a group to discuss implementation issues during the project. This might have been because they were expecting the researchers to manage the implementation given that was the aim of the research project. However, though support staff welcomed feedback from researchers about the impact of Check Up GP on young people’s experience of care, a number of these support staff expressed frustration at the lack of feedback and appreciation directly from the participating principal GPs. These findings suggest that there is a need for internal practice leadership to drive and support the implementation process in primary care; this is particularly important if the process is to succeed in the absence of external researchers.

Despite the challenges, GPs expressed a desire to continue using Check Up GP after the conclusion of the study. There was some discrepancy between GPs on how and when they wanted to use the tool in the future, for example, whether opportunistically in acute care as in this study or in a separate consultation. A separate appointment would be preferred by one GP, who was unwilling to run over time. Separate consultations for preventive screening may afford GPs more time but prior evidence shows low attendance of young people at dedicated preventive care well-child visits, particularly by the youth of low-income [27,28,63]. Furthermore, compared with planned well visits in the United States, dedicated to screening for health and lifestyle issues, opportunistic screening is more effective at detecting issues in young people [64]. This suggests that integrating a screening opportunity into young people’s routine acute care visits is the best way to maximize reach and detection. One option is for governments to subsidize a longer appointment to screen young people within routine consultations.

The willingness of support staff to continue to use the app sits in contrast to a previous study where receptionists developed negative views about administrating a paper-based alcohol screening tool to an adult population over time [65]. A number of important methodological features in our study may have contributed to the more positive views of support staff toward continuing the implementation of Check Up GP. These features include involving support staff in iteratively improving the tool, regularly informing them about the positive impact using the tool was having on young people’s experience of care, using a technology-based tool that was codesigned in part with input from support staff, and having researchers located at the clinic throughout the implementation period to troubleshoot administrative and technical issues. Given their unique and critical role and experience with this technology, support staff should be included not only in the codesign process of a tool which will affect their workflow but also in tailoring the implementation of a screening technology in primary care.

A particularly positive finding of our study was that using Check Up GP facilitated youth-friendly practice by making it easier for GPs to ask for consulting time alone with the adolescent. Having time alone with their GP is recommended as best practice for adolescent care [66], and young people who receive confidential care are more likely to discuss sensitive issues [67,68]. Despite this, the rates of young people seeing their GP alone are low [63,69]. Our findings suggest that the use of a health and screening app provides GPs with greater agency and confidence in asking for time alone and providing quality care.

Finally, our findings suggest that, not only users of a screening tool but parents attending with their child have the potential to undermine confidential care and influence the successful implementation of such a tool. Our GPs and support staff observed that many parents looked over the shoulder of their child when completing Check Up GP. This finding was reflected in the youth exit survey results where, although a minority, 7% (6/85) felt they did not have enough privacy completing Check Up GP. Privacy and confidential care is a core requirement of youth-friendly health care [70], so for a screening app to be useful and trusted it is essential that young people have sufficient privacy while using it. Future implementations of a screening tool need to ensure adequate privacy for young people, such as by providing an option of completing the app on their own smartphone, instead of a tablet, or by inviting them to use it on their own in a separate room. Another useful strategy for diverting parents’ attention away from their child completing the screening tool would be giving parents their own survey to complete about any assistance they may need, an option available through the American Academy of Pediatrics’ Bright Futures Guidelines [2].

### Limitations

This study had a number of limitations. We conducted a single case study, implementing the tool at one English-speaking volunteer clinic in an area of relative socioeconomic advantage with GPs who identified as being enthusiastic, knowledgeable, and confident in consulting with young people. So, it is possible that a different type of clinic, such as a community health clinic, one with a predominantly multilingual or sociodisadvantaged population, or less youth-friendly GPs, may implement and use a tool such as Check Up GP differently to the practice in this study. As such, our findings may not be generalizable.

Another limitation is that due to the relatively brief study length and limited staff availability, we were only able to conduct in-depth interviews of GPs and support staff at the end of the implementation period. Thus, our analysis provided rich insights at only one moment in time. Conducting interviews at a number of time-points through implementation may have provided further insights; however, recording the support provided to staff throughout the implementation period provided valuable insights over time.

### Future Research

Although different general practices share similarities, they are diverse in a number of important ways, such as in size, location, opening hours, degree of corporatization, billing practices (free universal national health insurance versus fee for service), salaried versus nonsalaried GPs, and patient sociodemographic characteristics. Thus, further research is needed to investigate the implementation of a health and lifestyle screening tool in a range of clinics. We recommend that future projects should also ensure that the app is integrated within existing clinical software to minimize the additional work required by support staff and GPs to use this new technology.

### Conclusions

The implementation of technology in time-restricted and dynamic settings such as general practice presents a range of technical and administrative challenges. Our study reveals new insights into the impact of integrating a health and lifestyle screening app into the routine care of young people on the roles and responsibilities of both GPs and support staff. We present a rich picture of the practical problems that can arise when screening tools are introduced into busy clinics and the solutions that GPs and support staff devise in response to them. Our findings will benefit future researchers and practitioners seeking to implement screening tools in real-world settings. Successful implementation of this technology is possible but requires adequate time, intensive facilitation, organizational leadership, and cycles of iteration. More resources, external to staff, are needed to drive and support sustainable technology innovation and implementation in general practice settings.

## Appendix

Multimedia Appendix 1 Interview schedule for interviews with general practitioners and practice support staff.

## Abbreviations

FTE: full time equivalent
GP: general practitioner
LGA: Local Government Area
NPT: normalization process theory
PDSA: Plan-Do-Study-Act
PM: practice manager
RC: reception coordinator
SMS: short message service
