# Supplementary material for: Experiences of General Practitioners and Practice Support Staff Using a Health and Lifestyle Screening App in Primary Health Care: Implementation Case Study
Source: JMIR Mhealth Uhealth. 2018 Apr 24;6(4):e105. doi: 10.2196/mhealth.8778 (PMC5941099; doi:10.2196/mhealth.8778)
Supplement: Multimedia Appendix 1 [file mhealth_v6i4e105_app1.pdf]

Multimedia Appendix 1: Interview schedule for interviews with General Practitioners and practice support staff

| General Practitioner | Practice support staff |
|---|---|
| 1. Can you talk about your experience of using the app with young people?<br>2. How did using the app differ from existing/previous practice with young people?<br>3. What were the benefits of using the app with young people?<br>    **a.** Did it have an impact on the way you communicated with young people?<br>    **b.** Did it raise any issues outside the scope of your usual consults with young people?<br>    **c.** Did it effect the communication with parents?<br>4. Were there any surprising or unexpected things about using it?<br>**5.** Did your opinion of using Check Up GP change over time?<br>6. What were some of the challenges in integrating Check Up GP into your practice?<br>    a. What are the possible solutions for these?<br>7. How did you have to adapt or change your work?<br>    b. Did it help or impede your work?<br>    c. How much extra work did it require from you?<br>    d. Did this change over time at all?<br>8. Would you see it as something you/the practice should continue to use in your regular practice with young people? What would it take to make this possible? | 1. Can you tell me in your words, what the purpose of Check Up GP app is?<br>2. What do you think were the benefits of using the app for young people? For GPs?<br>3. Can you talk about your experience of administrating the app?<br>    a. Did you have to adapt or change your work?<br>    b. Did it help or impede your work? How much extra work (time, additional tasks, learning to use it etc) did it require from you?<br>    c. Did this change over time at all?<br>4. What were some of the challenges in using the app?<br>    a. What are solutions for this?<br>5. Were there any surprising or unexpected things about your role in administrating it?<br>6. Would you see it as something the practice should continue to use?<br>**7.** Do you think administrating new technology like this fits under your job description?<br>**8.** Is there anything else you'd like to say about the app or ways to improve its administration in general practice? |
